# Pharmacological Characters and Toxicity Evaluation of Coumarin Derivative LP4C as Lead Compound against Biofilm Formation of *Pseudomonas aeruginosa*

**DOI:** 10.3390/molecules28073138

**Published:** 2023-03-31

**Authors:** Moxi Yu, Jiajia Xin, Yongsheng Liu, Yamiao Chen, Hui Zhao, Yaoyao Li, Yachen Hou, Min Jia, Bin Wang, Mingkai Li

**Affiliations:** 1 School of Pharmacy, Shaanxi University of Chinese Medicine, Xi’an 712046, China; 2 Department of Pharmacology, School of Pharmacy, Air Force Medical University, Xi’an 710032, China; 3 Department of Blood Transfusion, Xijin Hospital, Air Force Medical University, Xi’an 710032, China

**Keywords:** *Pseudomonas aeruginosa*, coumarin, biofilm, infection, lead compound

## Abstract

*Pseudomonas aeruginosa*-induced biofilm infection is difficult to treat and poses a significant threat to public health. Our previous study found a new coumarin derivative LP4C which exerted potent in vitro and in vivo anti-biofilm activity against *Pseudomonas aeruginosa*; however, the underlying molecular mechanism and drug-likeness of LP4C is unclear. In this study, we confirmed that LP4C could inhibit the biofilm in dose-dependent manner without bactericidal activity. The transcriptomic profiling and RT-PCR result revealed that bacterial pyrimidine mediated the inhibitory activity of LP4C. The cell viability was not affected in LP4C treatment groups with the concentration under 200 μg/mL, and no death or toxicity sign was observed in mice treated by 20, 40 and 80 mg/kg LP4C during the three-week test period. Ames test presented that LP4C had no effect on the bacterial reverse mutation. In additional, pharmacokinetic results showed that LP4C was likely to have the orally bioavailable properties. Our data indicate that LP4C is a possible lead compound for the development of new anti-biofilm infection agents against *Pseudomonas aeruginosa*.

## 1. Introduction

Bacteria in biofilm exhibit different properties than the free-living bacterial cell, including adaption, adherence and persistence [1]. It is reported that about 80% chronic infections are associated with bacterial biofilm, which are common in osteomyelitis, infective endocarditis, implant related infections, such as catheter-associated urinary tract infection, and chronic wound infection [2,3]. Although almost all bacteria are able to induce the biofilm in certain conditions, the biofilm elaboration is markedly enhanced in the *Pseudomonas aeruginosa* (*P. aeruginosa*), and causes ventilator-associated pneumonia, pulmonary cystic fibrosis, central venous catheterization and catheter related infections, as well as surgical and transplant infections [4,5]. In addition, *P. aeruginosa* becomes the major cause of hospital-associated infection and ventilator-associated pneumonia, usually due to the failure of anti-bacterial agent treatment [6,7], which poses a great challenge to infection control and treatment.

The bacterial biofilm is constructed by a matrix of extracellular polymeric substances (EPS) primarily consisting of proteins, lipids, polysaccharides and extracellular DNA (eDNA); the structural and functional diversity vary based on the physical state and bacterial species [8,9]. In *P. aeruginosa*, polysaccharides, such as Psl, Pel and alginate, are tremendously involved in the structural stability, which are beneficial for biofilm initiation and growth [5]. The eDNA and cytosolic proteins released from the cell lysis are also crucial constituents of *P. aeruginosa* biofilm; they not only function as a nutrient source, but also activate the type VI secretion system and influence the inflammatory process [10]. Additionally, during the development of *P. aeruginosa* biofilm, the bacteria use quorum sensing (QS) circuits, cyclic dimeric guanosine monophosphate (c-di-GMP) and RNA regulators to build intercellular communities [11,12]. Thus, understanding the molecular basis of bacterial communities will uncover the new target for biofilm-associated infection treatment.

Recently, increasing evidence has shown that plant-derived molecules exert anti-biofilm activity in *P. aeruginosa*. For example, shikonin from *Boraginaceae* species can inhibit the biofilm formation of *P. aeruginosa* PAO1 strain at 200 μM [13]. Furthermore, allicin and ajoene from *Allium sativum L.* inhibit EPS production, down-regulate the QS-related virulence factor of *P. aeruginosa* and improve the bacterial clearance in mouse infection models [14,15,16]. Coumarins are important heterocyclic molecular framework of bioactive molecules and the derivatives exit in the plants widely. Our previous study showed that 4-hydroxycoumarin derivate DCH could inhibit the biofilm formation of methicillin-resistant *Staphylococcus aureus* (MRSA) selectively [17]. Interestingly, we screened another coumarin derivate LP4C which exhibited significant inhibitory activity to biofilm formation in *S. aureus* and *P. aeruginosa* [18,19]. These results highlight the potential role of coumarin derivatives as new therapeutic agents to combat the bacterial biofilm infection.

In this study, we sought to explore the underlying mechanism of LP4C against biofilm infection caused by *P. aeruginosa*, and evaluate its safety and drug-likeness by cytotoxicity assay, acute toxicity test, AMS test and pharmacokinetic measurement. These results will be beneficial not only in searching for the potential therapeutic target for biofilm infection, but also for providing the basis to develop new therapy agents.

## 2. Results

### 2.1. LP4C Inhibits the P. aeruginosa Biofilm without Bactericidal Activity

**Figure 1:**
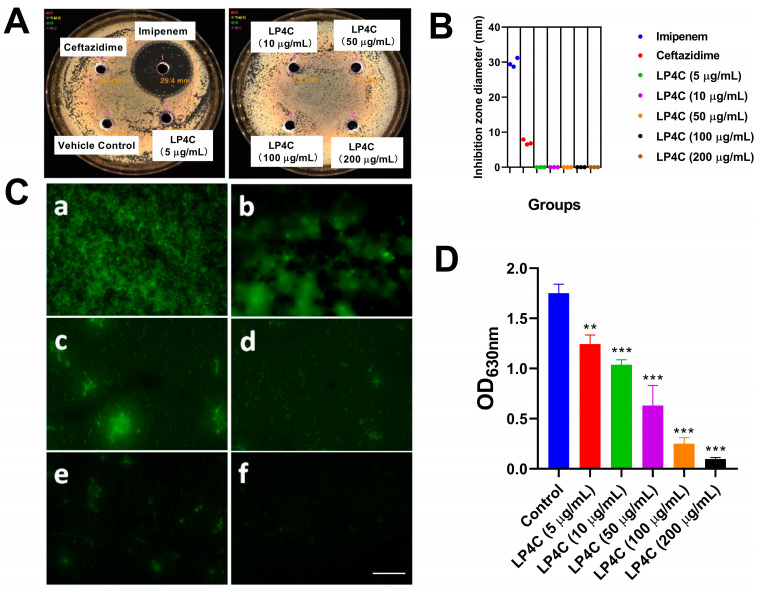
Inhibitory activity of LP4C to the *P. aeruginosa* biofilm without bactericidal activity. (**A**) Representive image of inhibition zone of LP4C (5, 10, 50, 100, 200 μg/mL) and control antibiotic imipenem (10 μg/mL) or ceftazidime (10 μg/mL) to the *P. aeruginosa*. (**B**) Analysis of inhibition zone diameter of LP4C (5, 10, 50, 100, 200 μg/mL), imipenem and ceftazidime. (**C**) Fluorescence staining of bacterial biofilm after vehicle control treatment (**a**) or 5 μg/mL (**b**), 10 μg/mL (**c**), 50 μg/mL (**d**), 100 μg/mL (**e**) and 200 μg/mL (**f**) LP4C treatment. (**D**) Crystal violet staining results showed that LP4C could inhibit the formation of the *P. aeruginosa* biofilm in a dose-dependent manner. ** *p* < 0.01, *** *p* < 0.001 vs. control, *n* = 3. Scale bar = 20 μm.

To observe the inhibitory potency of LP4C to the growth and biofilm formation of the *P. aeruginosa* (ATCC27853) strain, the bacteria were treated by LP4C (5, 10, 50, 100 200 μg/mL) and reference antibiotics imipenem (10 μg/mL) and ceftazidime (10 μg/mL); the inhibition zone diameter was then measured (Figure 1A). The result showed that imipenem could inhibit the growth of *P. aeruginosa* obviously, while LP4C and ceftazidime had no bactericidal activity (Figure 1B). Interestingly, the fluorescein isothiocyanate (FITC) staining showed that LP4C suppressed the formation of biofilm in a dose-dependent manner compared to the Vehicle Treatment Group (Figure 1C). This result was further confirmed by crystal violet staining; these results showed that LP4C ranging from 5 μg/mL to 200 μg/mL could inhibit the biofilm formation in a dose-dependent manner (Figure 1D).

### 2.2. Bacterial Pyrimidine Involved the Inhibitory Activity of LP4C

**Figure 2:**
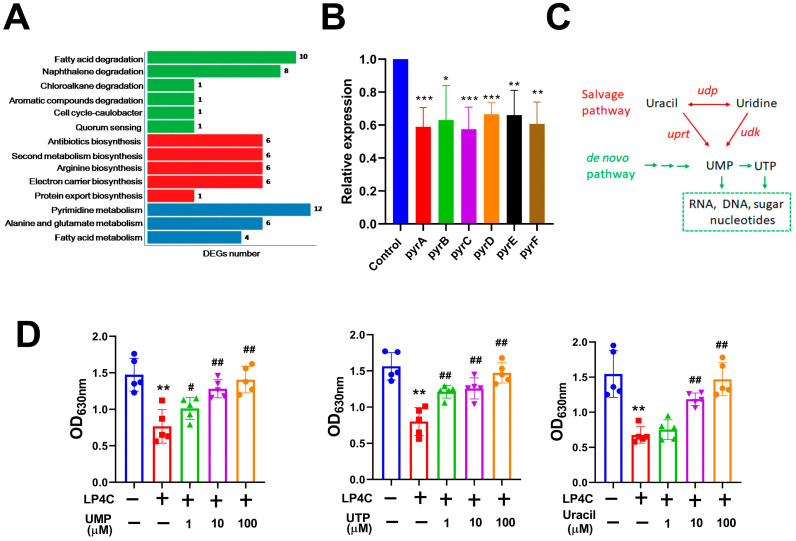
Pyrimidine involved in the inhibitory activity of LP4C. (**A**) The DEGs number of most enriched pathway for target genes after LP4C (10 μg/mL) treatment in *P. aeruginosa*. (**B**) Expression of *pyr* genes (*pyrA*, *pyrB*, *pyrC*, *pyrD*, *pyrE* and *pyrF*) in Control and LP4C (10 μg/mL) Treatment Groups. * *p* < 0.05, ** *p* < 0.01, *** *p* < 0.001 vs. control, *n* = 3. (**C**) The illustration of biosynthesis pathway of pyrimidine. (**D**) Exogenous pyrimidine reversed the inhibitory activity of LP4C to biofilm formation in *P. aeruginosa*. ** *p* < 0.01 vs. Untreated Control Group, ^#^  *p* < 0.05, ^##^  *p* < 0.01 vs. LP4C Treatment Group, *n* = 5.

To investigate the underlying molecular mechanism of LP4C against *P. aeruginosa* biofilm, the transcriptome assay was performed on *P. aeruginosa* (ATCC27853) strain. Compared to the Vehicle Treatment Group, LP4C (10 μg/mL) reduced the expression of many bioactive molecules involved in the pathway, such as fatty acid, naphthalene, antibiotics, arginine, electron carrier, protein export, pyrimidine, alanine and glutamate; the DEGs number of pyrimidine metabolism was the most enriched (Figure 2A). In the bacterial pyrimidine biosynthesis pathway, carbamoyl phosphate synthase (*pyrA*), aspartate transcarbamoylase (*pyrB*), dihydroorotase (*pyrC*), dihydroorotate dehydrogenase (*pyrD*), orotate phosphoribosyltransferase (*pyrE*) and orotidylic acid decarboxylase (*pyrF*) are the key enzymes; our RT-PCR results confirmed that LP4C could inhibit the expression of *pyr* genes significantly (Figure 2B). It was reported that both the salvage and *de novo* pathways were important for synthesis of bacterial pyrimidine [20]. Bacteria biosynthesizes the uridine monophosphate (UMP) and uridine triphosphate (UTP) through the *de novo* synthesis or salvage pathways; these pyrimidines contribute to the biosynthesis of RNA and DNA (Figure 2C). Interestingly, supplementation of uracil (10 and 100 μM) and UMP (1, 10 and 100 μM) or UTP (1, 10 and 100 μM) could reverse the biofilm formation ability impaired by 10 μg/mL LP4C (Figure 2D). This result indicated that the bacterial pyrimidine biosynthesis pathway was possibly involved in the molecular mechanism of LP4C.

### 2.3. Cytotoxicity and Acute Toxicity of LP4C

**Figure 3:**
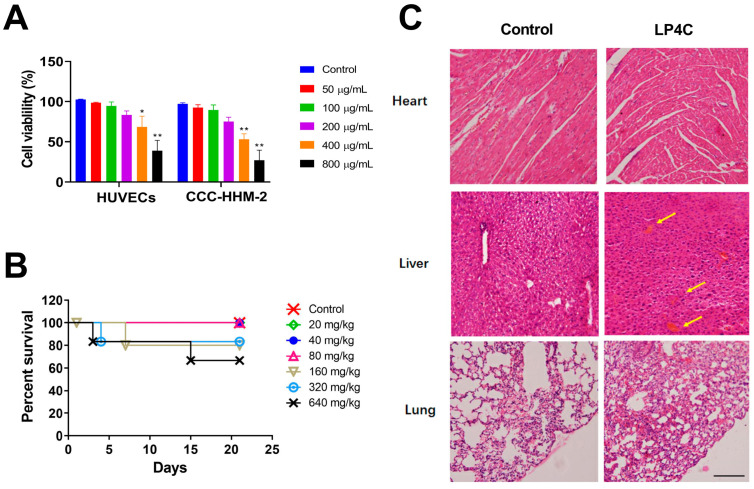
Observation of cytotoxicity and acute toxicity of LP4C. (**A**) Effect of LP4C on the viability of mammalian cells as determined by CCK-8 assay. * *p* < 0.05, ** *p* < 0.01 vs. Control Group, *n* = 6. (**B**) Percentage survival was measured after the three-week-period for intragastric administration of LP4C at doses of 20, 40, 80, 160, 320, and 640 mg/kg daily in mice, *n* = 10. (**C**) Pathological change observation of tissue sections stained with H&E; the slight hemorrhagic phenomena occurred in the liver tissue from 320 mg/kg LP4C Treatment Group (arrows). Scale bar = 200 μm.

As safety is essential throughout the spectrum of drug discovery and development, we observed the effect of LP4C on two human cell lines, including HUVEC (human umbilical vein endothelial cell) and CCC-HHM-2 (human embryonic myocardial tissue cell). As shown in Figure 3A, after treatment of LP4C (50, 100, 200, 400 and 800 μg/mL) for 24 h, in contrast to the Vehicle Treatment Control Group, the cell viability was not affected in LP4C treatment groups with the concentration under 200 μg/mL; this was at least 40 times higher than the anti-biofilm concentration of LP4C. This result implied that LP4C had much less toxicity to mammalian cells. Furthermore, after the intragastric administration of LP4C at doses ranging from 20 mg/kg to 640 mg/kg bodyweight in C57BL/6 mice, no death and obvious toxicity sign was observed in 20, 40 and 80 mg/kg LP4C treatment groups during the three-week period. The median lethal dose (LD_50_) is an indicator of a substance’s toxicity in the short term; the analysis showed that there was not a significant difference in the survival rate between the Control and LP4C Treatment Groups ranging from 20 mg/kg to 320 mg/kg, while the LD_50_ of LP4C was 1.352 g/kg (Figure 3B). The vital organs, including heart, liver, lung, kidney and spleen, were excised and the histopathological changes were observed after H&E staining; in contrast to the control groups, no obvious pathological change was observed, except the slight hemorrhagic phenomena that occurred in the liver section in the 320 mg/kg LP4C Treatment Group, which was indicated by yellow arrows (Figure 3C). Thus, the acute toxicity study suggests that acute exposure to compound LP4C is relatively safe in the mammalian.

### 2.4. The Bacterial Reverse Mutation Assay of LP4C

Next, we use the bacterial reverse mutation assay (Ames assay) to determine the mutagenic potential of LP4C with *Salmonell typhimurium* (*S. typhimurium*) strains TA97, TA98, TA100 and TA102. In contrast to the DMSO Solvent Negative Control (100 μL/plate) Group, the number of bacterial revertant colonies did not increase in LP4C treatment groups ranging from 50 μg/plate to 5000 μg/plate. Meanwhile, there were a large number of revertant colonies in positive control groups in comparison to the Negative Control Group and all LP4C treatment groups (Table 1). This result indicates that LP4C will not cause mutation in *S. typhimurium* test strains and has no obvious mutagenic potential.

### 2.5. Pharmacokinetics and ADME Properties of LP4C

**Figure 4:**
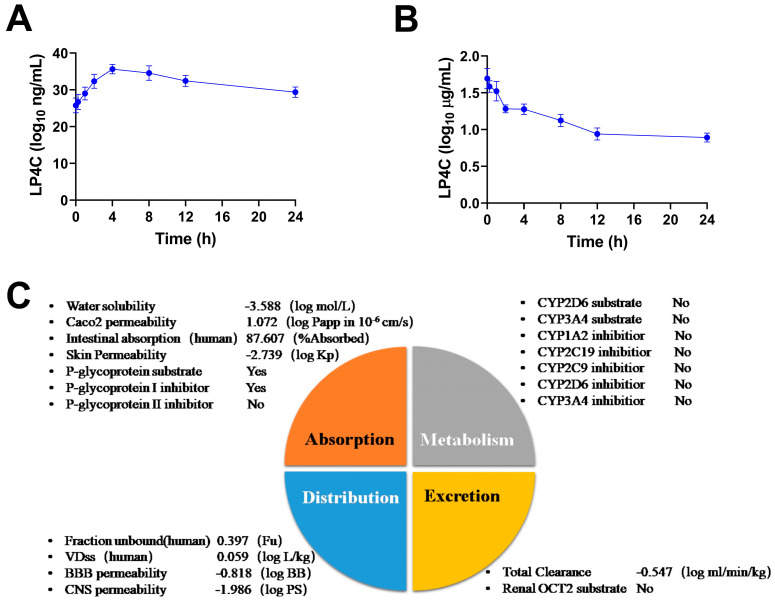
Pharmacokinetic character and ADME properties of LP4C. Quantification of LP4C serum concentration in rat blood after intragastric administration at 10 mg/kg dosage (**A**) or intravenous administration at 5 mg/kg dosage (**B**), *n* = 5. Prediction of ADME properties of LP4C conducted through graph-based signatures (**C**).

To investigate the pharmacokinetic character of compound LP4C, single-dose intragastric administration of LP4C (10 mg/kg) or intravenous administration of LP4C (5 mg/kg) was performed on SD rats. The results showed that the blood concentration of LP4C increased within the initial 4 h after the intragastric administration, with a plasma half-life of 11 ± 1.26 h (Figure 4A); the serum concentration of LP4C then declined rapidly over the initial 10–60 min after intravenous administration, with a plasma half-life of 14 ± 3.05 h (Figure 4B) and bioavailability of LP4C of 46.35%. The absorption, distribution, metabolism and excretion (ADME) studies of LP4C were conducted through graph-based signatures, which predicted ADME of small molecular compounds. The results indicated that LP4C had a high Caco-2 cell permeability with the predicted values at 1.072 × 10^−6^ cm/s, while the volume of distribution (VDss) was low with the log value at 0.059 L/kg. Meanwhile, LP4C might not be the substrate of cytochrome P450 enzymes CYP2D6 and CYP3A4 or the inhibitor of CYP1A2, CYP2C9, CYP2C19 and CYP2D6. More detailed information is shown in Figure 4C. According to Lipinski’s rule of five, including the molecular weight, LogP, rotatable bonds, hydrogen acceptors and donors, LP4C is likely to have the required orally bioavailable chemical and physical properties (Supplementary Table S1).

## 3. Discussion

Coumarins are important phenolic substances and exhibit the broad-spectrum anti-biofilm activity against a wide range of bacteria [21,22,23]. Our previous results showed that coumarin derivative LP4C was the potential anti-bacterial biofilm compound in vitro and in vivo [18,19]. The present study further confirmed that LP4C inhibited bacterial biofilm formation even at 5 μg/mL, which was about 80 times lower than the other reported coumarin derivatives, such as esculetin (6,7-dihydroxycoumarin) and esculin [24]. Interestingly, due to the fact that LP4C has no bactericidal activity against *P. aeruginosa*, it will not pose antibiotic pressure to the bacteria.

Biofilm formation in *P. aeruginosa* is a complicated process involving multiple signals and regulatory networks. The transcriptome result revealed that LP4C could inhibit the expression of genes related to the bacterial metabolism pathway most significantly. It was reported that the regulation of pyrimidine biosynthesis in *P. aeruginosa* differed from the *Pseudomonas* species, and the pyrimidine synthetic activity in *Pseudomonas* species was active at varying levels [24,25]; this agreed with what we observed in this study. Since the enzymes involved in the bacterial pyrimidine salvage biosynthetic pathway considerably differ from those present in humans [26], they might be attractive targets for biofilm infection therapy.

At present, the function of pyrimidine involved in biofilm formation and maintenance is not fully understood. In *Escherichia coli*, inactivation of genes related to the *de novo* pyrimidine biosynthetic pathway inhibited transcription of the csgDEFG operon and biofilm master regulator CsgD protein, reduced the production of cellulose and curli fibers; supplementing the exogenous pyrimidine in the culture media also restored the production of curli [27]. In consistent with this finding, our results showed that the supplement of uracil, UMP or UTP reversed the inhibition of compound LP4C to the bacterial biofilm formation. Additionally, it was confirmed that uracil influenced the LasR, RhIR and PQS quorum sensing system and further repressed the biofilm formation of *P. aeruginosa* [28]. From this perspective, the results from our team and other labs indicate that pyrimidine mediates the bioformation of bacterial biofilm; the agent targeting bacterial pyrimidine metabolism pathway has a potential for the development of biofilm infection therapeutic drugs.

The unique heterocyclic structure makes coumarin derivative a privileged scaffold in medicinal chemistry; it also exhibits versatile pharmacological properties [29,30], some of which are used frequently in clinical tests. For example, 4-hydroxycoumarin anticoagulants typified by warfarin inhibit the function of vitamin K and exert the anticoagulant activity [31]. Aminocoumarin antibiotic Novobiocin inhibits bacterial DNA gyrase and heat shock protein in cancer cells and is used as an antibacterial and anticancer agent [32,33]. Meanwhile, the pharmaceutical properties and toxicity of the coumarins vary significantly based on their specific chemical structure; it was reported that coumarin derivatives could induce hepatocellular dysfunction and possessed an inhibitory effect on human CYP enzymes [34,35]. In this study, the obvious cytotoxicity and acute toxicity of LP4C was not observed, and the pharmaceutical profile indicated that LP4C had no inhibitory activity on the CYP enzymes, including CYP1A2, CYP2C19, CYP2C9 and CYP2D6. Additionally, the chemical properties of LP4C fulfill Lipinski’s rule of five, strengthening its potential as a promising candidate for drug development.

## 4. Materials and Methods

### 4.1. Chemicals

The coumarin derivative LP4C was synthesized according to the method reported previously [19]. The infrared spectra of LP4C were determined by a spectrophotometer (Bruker Fourier, Ettlingen, Germany). ^1^H Nuclear Magnetic Resonance (NMR) spectrum, ^13^C NMR spectrum, DEPT-135 NMR spectrum, ^1^H-^1^H COSY, Heteronuclear Single Quantum Coherence (HSQC) and Heteronuclear Multiple-Bond Correlation (HMBC) characterization data were obtained using the spectrometer (Varian Inova-500, CA, USA), which is shown in Supplementary Figure S1 and Table S2. Samples of 4-nitrobenzene-1,2-diamine, mitomycin C, 2-aminofluorene and 1,8-dihydroxyanthraquinone were purchased from Sigma-Aldrich (St. Louis, MO, USA).

### 4.2. Bacterial Strain and Animals

*P. aeruginosa* (ATCC27853) was purchased from the American Type Culture Collection, while *S. typhimurium* test strains TA97, TA98, TA100 and TA102 were obtained from Xi’an Guorui drug safety evaluation center. C57BL/6 mice and SD rats were obtained from the Lab Animal Centre of the Air Force Medical University. The animal experiment was approved by the Animal Use Ethics Committee of the Air Force Medical University.

### 4.3. Anti-Bacterial Activity Measurement

*P. aeruginosa* (ATCC27853) was cultured in 3 mL LB culture medium, shaken at 220 rpm for 12 h at 37 °C and diluted to 1 × 10^6^ CFU/mL. The culture disk was coated with 1% agar, and 100 μL bacterial suspension was added to the agar in the disk evenly. After the four holes were punched on the agar of each disk, the compound LP4C and imipenem were solved with DMSO and diluted to the final concentrations (5, 10, 50, 100 and 200 μg/mL LP4C and 10 μg/mL imipenem) in the PBS; ceftazidime was also diluted to 10 μg/mL in the PBS. LP4C or reference antibiotics were then added in each hole. The disk was cultured for 12–16 h at 37 °C; the result was then observed and the inhibition zone diameter was measured.

### 4.4. Growth and Measurement of Bacterial Biofilm

The bacterial biofilm growth and measurement was performed according to our previously reported method [17,19]. Briefly, *P. aeruginosa* (ATCC27853) was cultured in the tube with nutrient broth containing 2% (*w*/*v*) glucose, shaken at 220 rpm for 12 h at 37 °C. The suspension was diluted to 1 × 10^6^ CFU/mL and added to 96-well plates, the bacteria was cultured for 24 h at 37 °C for biofilm growth and the planktonic bacterial of each well was removed by PBS rinsing three times. To observe the biofilm formation, the fluorescein isothiocyanate (FITC) was added into the plates and incubated for 2 h at 37 °C; the labeled biofilm was then washed by PBS and visualized under the fluorescence microscopy (Olympus CKX53, Tokyo, Japan). The measurement of biofilm was performed according to the following procedure: the mature biofilm was fixed with 150 μL methanol and left to air dry, then stained with 150 μL of 1% (*w*/*v*) crystal violet solution for 15 min at room temperature. The plate was rinsed with PBS three times and 150 μL of glacial acetic acid was added to each well; the optical density was measured at 630 nm optical density using a microplate reader (Bio-Tek ELX800, Berten, USA).

### 4.5. RNA Isolation and RNA-seq

According to the previously described method [36], total RNA was extracted from the control or 10 μg/mL LP4C treatment *P. aeruginosa* (ATCC27853) group samples; each group contained three biological replicates. The RNA sequencing was performed using Illumina HiSeq4000 according to the operation instructions (Illumina, CA, USA). Differential gene expression analysis of LP4C treatment and control groups was conducted; KEGG signaling pathway analysis was performed to map the connection of multiple genes.

### 4.6. Quantitative Real-Time PCR (qPCR)

The total RNA was isolated from *P. aeruginosa* cultures, and qPCR amplification was performed according to the instrument of the quantitative PCR system (Stratagene Mx3005P, Agilent, USA); the thermal cycler was then initiated by denaturation procedure for 2 min at 93 °C followed by 40 cycles of 1 min at 93 °C and 2 min at 55 °C. The gene-specific PCR primers were listed in Supplementary Table S3. The relative expression of genes was analyzed by the 2^−ΔΔCt^ method with normalization to *gyrB* level.

### 4.7. Cytotoxicity Test

The cytotoxicity of LP4C to the human HUVECs and CCC-HHM-2 cells was tested by the cell counting kit-8 (CCK-8) staining. Briefly, cells (5 × 10^3^ cells per well) were cultured in confluent 96-well plate for 12 h, before LP4C at various final concentrations (50, 100, 200, 400, 800 μg/mL) was added into each well for 24 h; after 10 μL CCK-8 was added into the culture medium, the plate was incubated for 2 h at 37 °C. Optical density was calculated by the microplate reader (BioTek flx800, Berten, USA).

### 4.8. Acute Toxicity Assay

C57BL/6 mice were randomly divided into seven groups (10 mice/group), including the Vehicle Control Group and intragastric administration of LP4C groups (20, 40, 80, 160, 320 and 640 mg/kg bodyweight). All animals were closely observed every day for three weeks. The body weight and toxic effects were monitored and the survival rate was calculated. At the end of experiment, the survival mice were euthanized; vital organs, including heart, liver, lung, kidney and spleen, were then excised, weighed and preserved for histopathological observation.

### 4.9. Ames Test

Suspension of *S. typhimurium* test strains was prepared and incubated on the agar plates with the different concentrations of LP4C (50, 200, 1000, 2000, 5000 μg/plate), negative control DMSO vehicle (100 μL/plate) and positive control chemicals. In positive control groups, 4-nitrobenzene-1,2-diamine (20 μg/plate) was used for TA97, TA98, TA100 −S_9_ test strains, while mitomycin C (0.5 μg/plate) was used for for TA102 −S_9_ test strain. A sample of 2-aminofluorene (10 μg/plate) was used for TA97, TA98, TA100 +S_9_ test strains, while 1,8-dihydroxyanthraquinone (60 μg/plate) was used for the TA102 +S_9_ test strain. The histidine was added with the same amount. The plates were incubated for 48 h at 37 °C; the number of colonies was then counted per plate.

### 4.10. Pharmacokinetics Measurement

SD rats were orally administered with LP4C at 10 mg/kg or intravenously administered at 5 mg/kg; the blood samples were collected at different time points (10 min, 30 min, 1 h, 2 h, 4 h, 8 h, 12 h or 24 h after administration) from the caudal vein and the samples were centrifuged immediately. The concentration of LP4C in supernatant was measured by the liquid chromatography system (Aglilent 1100, Santa Clara, CA, USA).

### 4.11. ADME Properties Analysis

The graph-based signature was used for predicting the pharmacokinetic and toxicity properties of LP4C according to the reported pkCSM method [37]. The utility of the platform implemented was available in a freely accessible web interface (http://structure.bioc.cam.ac.uk/pkscm (accessed on 18 November, 2022); using this platform, the experimental analysis and optimization of absorption, distribution, metabolism and excretion (ADME), pharmacokinetic properties and possible toxicity of small molecules were performed.

### 4.12. Statistical Analysis

All values were expressed as mean ± SD. The data was analyzed by GraphPad Prism 8.1 software (Graph Pad, USA). The statistical significance was determined using one-way or repeated measure analysis of variance (ANOVA) and paired Student *t*-tests; the differences between survival rates were analyzed by Log-rank test and *p* < 0.05 was considered of statistical significance.

## 5. Conclusions

In summary, our results revealed that the bacterial pyrimidine metabolism pathway mediated the inhibitory activity of coumarin derivative LP4C against *P*. *aeruginosa* biofilm, and this compound had no obvious cytotoxicity, acute toxicity and mutagenic potential. Pharmacokinetic results showed that LP4C had the required orally bioavailable chemical and physical properties. However, success in uncovering the underlying mechanism of LP4C is still limited, and more safety evaluations, including long-term toxicity, need to be performed. These results indicate that coumarin derivative LP4C is a potential lead compound for the new biofilm infection agent against *P. aeruginosa* and propose the therapeutic strategy against biofilm infection.

## 6. Patents

The patent resulting from the work titled “Application of one pyrancoumarin derivative as anti-biofilm agent against *Pseudomonas aeruginosa*” was approved by the China National Intellectual Property Administration (ZL 201910317077.4).

## Figures and Tables

**Table 1:** The bacterial reverse mutation result of LP4C (mean ± SD, *n* = 3).

Group (μg/plate)	TA97		TA98		TA100		TA102	
	−S_9_	+S_9_	−S_9_	+S_9_	−S_9_	+S_9_	−S_9_	+S_9_
LP4C 5000 μg/plate	112 ± 9.83	141 ± 7.65	24 ± 3.5	31 ± 5.2	122 ± 7.67	130 ± 5.69	256 ± 9.21	273 ± 12.6
LP4C 2000 μg/plate	120 ± 8.53	140 ± 6.47	26 ± 6.7	32 ± 5.8	120 ± 5.71	132 ± 6.95	262 ± 8.44	280 ± 11.3
LP4C 1000 μg/plate	119 ± 8.52	142 ± 5.96	25 ± 5.4	32 ± 4.7	120 ± 8.33	131 ± 6.75	267 ± 8.35	279 ± 10.6
LP4C 200 μg/plate	122 ± 7.42	140 ± 8.55	26 ± 4.8	33 ± 4.1	116 ± 6.42	133 ± 7.34	271 ± 5.68	281 ± 9.36
LP4C 50 μg/plate	123 ± 5.92	142 ± 7.13	26 ± 2.2	32 ± 3.6	117 ± 5.96	130 ± 7.68	260 ± 10.3	276 ± 11.2
DMSO 100 μL/plate	122 ± 8.44	139 ± 8.26	24 ± 3.4	32 ± 3.8	119 ± 5.83	130 ± 4.58	261 ± 12.1	280 ± 8.87
Positive control	2014 ± 105	1354 ± 135	2027 ± 126	2036 ± 124	1376 ± 206	1273 ± 195	1673 ± 215	954 ± 63

## Data Availability

Data are contained within the article and Supplementary Materials.

## Supplementary Materials

The following supporting information can be downloaded at: https://www.mdpi.com/article/10.3390/molecules28073138/s1, Supplementary Figure S1: ^1^H NMR spectra, ^13^C NMR spectra, DEPT135 spectra, ^1^H-^1^H COSY, HSQC, and HMBC characterization data of LP4C; Supplementary Table S1: molecule properties of LP4C; Supplementary Table S2: the identification of ^13^C-NMR, DEPT135 and HSQC of LP4C; Supplementary Table S3: Primers of RT-PCR.

## Sample Availability

Samples are available from Moxi Yu.
