# Supplementary material for: Pharmacological Characters and Toxicity Evaluation of Coumarin Derivative LP4C as Lead Compound against Biofilm Formation of Pseudomonas aeruginosa
Source: Molecules. 2023 Mar 31;28(7):3138. doi: 10.3390/molecules28073138 (PMC10096344; doi:10.3390/molecules28073138)
Supplement: Supplementary file 1 [file molecules-28-03138-s001.zip › molecules-2288535-supplementary.pdf]

molecules

Supplementary Materials

Pharmacological Characters and Toxicity Evaluation of Coumarin Derivative LP4C as Lead Compound against Biofilm Formation of *Pseudomonas aeruginosa*

Moxi Yu[1,2,†], Jiajia Xin [3,†], Yongsheng Liu [2,†], Yamiao Chen[2], Hui Zhao [2],Yaoyao Li [2], Yachen Hou[2], Min Jia[2], Bin Wang [1,*] and Mingkai Li [2,*]

1 School of Pharmacy, Shaanxi University of Chinese Medicine, Xi'an 712046, China.
2 Department of Pharmacology, School of Pharmacy, Air Force Medical University, Xi'an 710032, China.
3 Department of Blood Transfusion , Xijin Hospital , Air Force Medical University, Xi'an 710032, China.
* Correspondence: mingkai@fmmu.edu.cn; Tel.: 086-187-0680-0326(M.L.) ; wangbin812@126.com (B. W.)
† These authors contributed equally to this work.

Supplementary Table S1. Molecule properties of LP4C.

Properties	Lipinski's Rule	Compound LP4C
Molecular weight	Less than 500	453.0665
LogP	Not greater than 5	3.5418
Rotatable bonds	Forward	2
Hydrogen Acceptors	No more than 10	5
Hydrogen Donors	No more than 5	3

Supplementary Table S2. The identification of ^{13}C-NMR, DEPT135 and HSQC.

serial number of atom	δ_C (ppm)	DEPT135	HSQC
12	37.07	CH	H-12
11	57.04	-	-
20, 21	123.79（q）	-	-
16, 18	130.68（q）	-	-
17	124.54（t）	CH	H-17
15, 19	129.48	CH	H-15, H-19
14	147.13	-	-
10	158.52	-	-
9	154.81	-	-
5	152.80	-	-
7	160.23	-	-
6	113.66	-	-
4	117.06	CH	H-4
1	123.19	CH	H-1
3	133.53	CH	H-3
2	125.09	CH	H-2
13	119.34	-	-
8	102.48	-	-

Supplementary Table S3. Primers of RT-PCR.

Gene		Primer sequence (5′-3′)
pyrA	Forward	CGTGCCGTTCGTCTCCAAGTG
	Reverse	GCGGGATGATTTCCTGGGTGAAG
pyrB	Forward	ACCGTGGCGTGGAGATCGAG
	Reverse	GCTGGCGTTGGGTGTTCTGG
pyrC	Forward	AACTCGGACTCTGGCGTGACC
	Reverse	GTGCTCGTCGATGAACTGCTTCTC
pyrD	Forward	GGTGCCTTTGCGTTTACAGATGAC
	Reverse	TCACAGTGTGCTACGATGGCTTTG
pyrE	Forward	GCGGTGATCGACAGCGGAATC
	Reverse	GTGGTCCTTGGCTTCCTTGCG
pyrF	Forward	CGACCTGAAATTCCACGACATCCC
	Reverse	GTTGACCATCCACACGCCCATC
gyrB	Forward	ACAGGAATCGGTGGCGACTTTG
	Reverse	GCTCCATCCACATCGGCATCAG

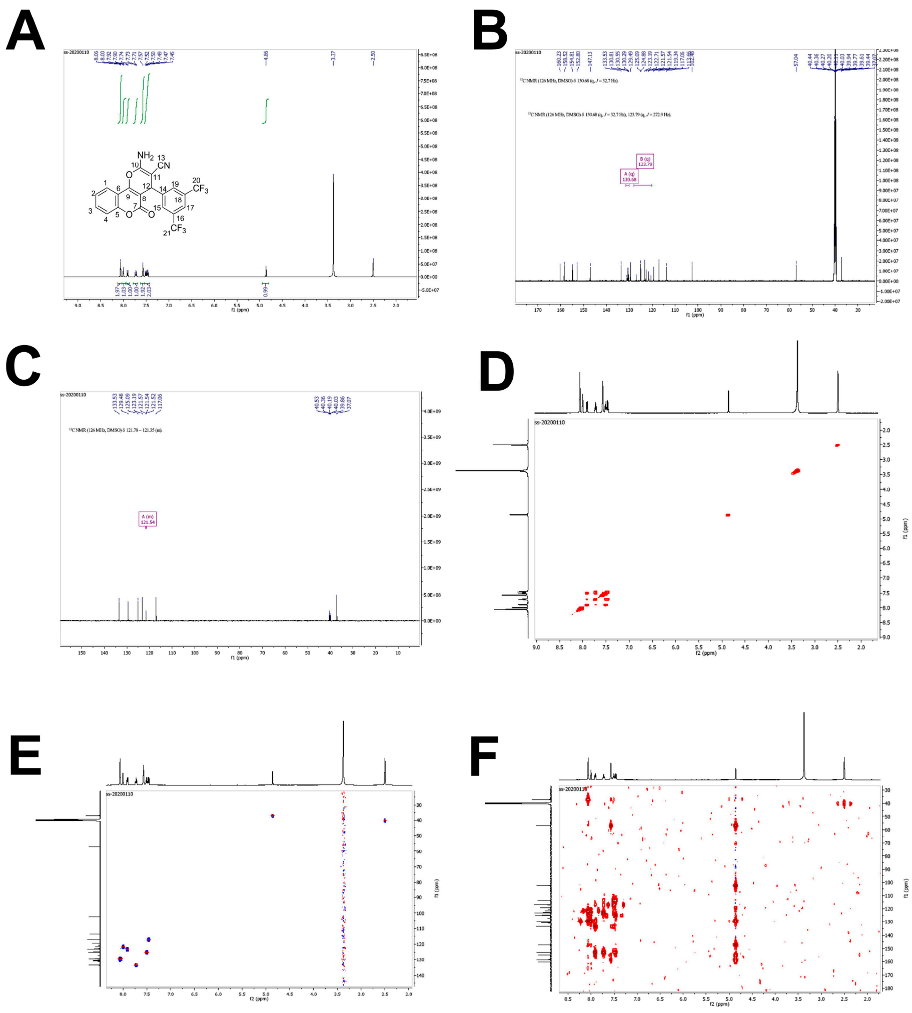

Supplementary Figure S1. The chemical structure identification of LP4C. The ^{1}H NMR spectra (A), ^{13}C NMR spectra (B), DEPT135 spectra (C), ^{1}H-^{1}H COSY (D), HSQC(E) and HMBC (F) characterization of LP4C.
